# Chimeric antigen receptor T-cell therapy for aggressive B-cell lymphomas

**DOI:** 10.3389/fonc.2024.1394057

**Published:** 2024-07-01

**Authors:** Bei Hu, Victoria Korsos, M. Lia Palomba

**Affiliations:** ^1^ Department of Hematologic Oncology and Blood Disorders, Atrium Health Levine Cancer Institute/Wake Forest School of Medicine, Charlotte, NC, United States; ^2^ Cellular Therapy Service, Department of Medicine, Memorial Sloan Kettering Cancer Center, New York, NY, United States; ^3^ Lymphoma Service, Department of Medicine, Memorial Sloan Kettering Cancer Center, New York, NY, United States

**Keywords:** chimeric antigen receptor T cell therapy (CAR T cell therapy), diffuse large B cell lymphoma (DLBCL), high grade B cell lymphoma, mantle cell lymphoma (MCL), relapsed and refractory lymphoma

## Abstract

Chimeric antigen receptor (CAR) T-cell therapy is a revolutionary approach in the treatment of lymphoma. This review article provides an overview of the four FDA-approved CAR T-cell products for aggressive B-cell lymphoma, including diffuse large B-cell lymphoma and mantle cell lymphoma, highlighting their efficacy and toxicity as well as discussing future directions.

## Introduction

Aggressive B-cell lymphomas are a heterogenous group of cancers arising from B lymphocytes that are typically fatal without treatment. The most common is diffuse large B-cell lymphoma (DLBCL), which is cured with rituximab and anthracycline-based chemoimmunotherapy in over 60% of patients. However, those with primary refractory disease, early relapse, or relapse after autologous stem cell transplant (ASCT) have a dismal prognosis with overall survival measured in months, based on the SCHOLAR-1 study (1). Although relatively indolent in some cases, mantle cell lymphoma (MCL) is typically aggressive, and while most patients respond well to frontline chemoimmunotherapy, all patients eventually relapse. Patients who progress on Bruton’s tyrosine kinase (BTK) inhibitor survive a median of 3–11 months (2–5). A more recent study for patients with relapsed or refractory MCL who progressed on BTK inhibition in the pre-CAR T-cell era is the retrospective SCHOLAR-2 study, which showed that the median overall survival (OS) from initiation of the first post-BTK inhibition therapy was 9.7 months (6).

Chimeric antigen receptor T-cell (CAR T) therapy has been one of the most revolutionary treatments for hematologic malignancies that have not responded to conventional therapy. CD19-directed CAR T-cell therapy is a type of immunotherapy that uses genetically modified T cells to target and kill cancer cells that express CD19, a protein found on most B-cell lymphomas. Herein we will review the currently Food and Drug Administration (FDA)-approved CAR T-cell therapies for DLBCL and MCL as of Jan 2024 and discuss the management of its toxicities.

## CAR T-cell therapy

CAR T-cell therapy is a multi-step process. Initially, the patient undergoes collection of autologous T cells through a process called leukapheresis. The cells are then shipped to the manufacturing site. CAR T cells are made by transduction of an inactivated viral vector into the patient’s autologous T cells to express proteins called chimeric antigen receptors (CARs) which then recognize and bind to the CD19 proteins expressed on the patient’s lymphoma cells. The final CAR T-cell product consists of the CD19 antigen domain, transmembrane spacer, a co-stimulatory domain, and finally the CD3ζ intracellular signaling domain (7). It is the presence of the co-stimulatory domains, in addition to the primary signal through the T-cell receptor, that results in full T-cell activation and CAR T-cell expansion and persistence, allowing for the improved efficacy of second-generation CAR T-cell therapy over the first-generation CARs (8). The type of co-stimulatory domain (CD28 or 4–1BB) is what accounts for the differences in toxicity in CAR T-cell products (9), with the CD28 co-stimulatory domain being associated with rapid and high peak expansion, thus resulting in more severe toxicities earlier on when compared with the 4–1BB costimulatory domain (10, 11). The manufacturing process takes 3–5 weeks depending on the product. During this time, patients may or may not receive bridging therapy in the form of systemic therapy or radiation to control the lymphoma. The cells are then shipped back to the treatment center, and prior to infusion of the CAR T cells, the patients receive lymphodepleting chemotherapy, which creates a favorable immune environment for CAR T-cell expansion and efficacy (12, 13). After the infusion of CAR T cells, the patients were then monitored for toxicity.

## FDA-approved CAR T-cell products

### Diffuse large B-cell lymphoma

#### Axicabtagene ciloleucel

Axicabtagene ciloleucel (axi-cel) is generated by using a retro-viral vector and includes a CD28 transmembrane domain and a CD28 co-stimulatory domain. Axi-cel was the first CAR T-cell therapy approved for relapsed/refractory (R/R) DLBCL, high-grade B-cell lymphoma, primary mediastinal B-cell lymphoma, and transformed follicular lymphoma after the failure of two lines of therapy based on the results of the phase 1/2 ZUMA 1 trial (**Table 1**) (14). Of the 111 patients enrolled in the study, product was successfully manufactured in 110 patients and infused in 101 patients. Bridging therapy was not allowed in the study. The median time from leukapheresis to the delivery of cells was 17 days (**Table 2**). The overall response rate (ORR) was 82%, with 54% achieving a complete response (CR). At median follow-up of 15.4 months, 40% of the patients continued to be in CR. The OS at 18 months was 52%. When compared with historical control with ORR of 26% (CR of 7%) and a median OS of 6.3 months as described in the SCHOLAR-1 study (1), the results of the ZUMA-1 trial were practice-changing and thus led to the approval of the first gene-based therapy by the FDA for large B-cell lymphoma. In a longer follow-up study with a median follow-up of 63.1 months, the median OS was 25.8 months, with estimated 5-year OS of 42.6% and disease-specific survival of 51% (15). The median duration of CR was 62.2 months and of those who achieved CR, the median OS was not reached with 5-year OS of 64%, supporting the curative potential of axi-cel.

**Table 1:** CAR T-cell therapy in aggressive B-cell lymphomas: clinical trials.

Drug	Trial	Indication	Dose	Bridging therapy (% received)
Axicabtagene ciloleucel	ZUMA-1 (14, 15) ZUMA-7 (16)	R/R DLBCL after 2 failed lines of therapy Primary refractory or relapsed DLBCL within 12 months	2 × 10^6^/kg 2 × 10^6^/kg	No Limited to steroids (36%)
Tisagenlecleucel	JULIET (17, 18) BELINDA (19)	R/R DLBCL after 2 failed lines of therapy Primary refractory or relapsed DLBCL within 12 months	0.6 to 6 × 10^8 ^ 0.6 to 6 × 10^8^	Yes (92%) Yes (83%)
Lisocabtagene maraleucel	TRANSCEND NHL 001 (20, 21) TRANSFORM (22) PILOT (23) TRANSCEND NHL 001 MCL cohort (24)	R/R DLBCL after 2 failed lines of therapy Primary refractory or relapsed DLBCL within 12 months Primary refractory or relapsed DLBCL transplant ineligible R/R MCL	50 to 110 × 10^6 ^ 90 to 110 × 10^6 ^ 90 to 110 × 10^6 ^ 50 to 100 × 10^6^	Yes (59%) Yes (63%) Yes (52%) Yes (66%)
Brexucabtagene autoleucel	ZUMA-2 (25, 26)	R/R MCL	2 × 10^6^/kg	Yes (37%)

CAR T cell, chimeric antigen receptor T cell; DLBCL, diffuse large B-cell lymphoma; MCL, mantle cell lymphoma.

**Table 2:** Efficacy of CAR T-cell therapy in aggressive B-cell lymphoma.

Trial		Ratio	Time (days)	ORR; CR	Median outcomes (months)	Long-term outcomes
ZUMA-1 (14, 15)		#infused/#leukapheresed: 62/74	Leukapheresis to delivery: 17	82%; 58%	PFS: 5.9 OS: 25.8	5-year PFS: 31.8% 5-year OS: 42.6%
ZUMA-7 (16)		#infused/#leukapheresed: 170/178	Leukapheresis to delivery: 13	ORR: axi-cel vs. SOC: 83% vs. 50% CR: axi-cel vs. SOC: 65% vs. 32%	Median EFS axi-cel vs. SOC: 8.3 vs. 2.0 HR = 0.4 95% CI 0.31 to 0.51 Median OS axi-cel vs. SOC: NR vs. 35.1 HR = 0.73 95% CI 0.53 to 1.01	2 year EFS axi-cel vs. SOC: 41% vs. 16% 2 year OS axi-cel vs. SOC: 61% vs. 52%
JULIET (17, 18)		#infused/#enrolled: 111/165	Enrollment to infusion: 54	52%; 40%	PFS: 2.9 OS: 11.1	
BELINDA (19)		#infused/#assigned: 155/162	Leukapheresis to infusion: 52	ORR: tisa-cel vs. SOC at week 12: 46% vs. 42% CR: tisa-cel vs. SOC at week 12: 28% vs. 27.5%	Median EFS tisa-cel vs. SOC: 3 months for both HR = 1.1 95% CI 0.8 to 1.4	
TRANSCEND NHL 001 (20, 21)		#infused/#leukapheresed: 294/344	Leukapheresis to delivery: 24	73%; 53%	DOR: 23.1 PFS: 6.8 OS: 27.3	2 year DOR: 49.5% 2 year PFS: 40.6% 2 year OS: 50.5%
TRANSFORM (22)		#infused/#leukapheresed (randomized to liso-cel): 90/92	Leukapheresis to product availability: 26	ORR liso-cel vs. SOC: 86% vs. 48% CR liso-cel vs. SOC: 66% vs. 39%	EFS: liso-cel vs. SOC: 10.1 vs. 2.3 HR = 0.35 95% CI 0.23 to 0.53 PFS: liso-cel vs. SOC: 14.8 vs. 5.7 HR = 0.41 95% CI 0.25 to 0.66 OS: liso-cel vs. SOC: NR vs. 16.4 HR = 0.51 95% CI 0.26 to 1.00	1 year EFS liso-cel vs. SOC: 44.5% vs. 23.7% 1 year PFS liso-cel vs. SOC: 52.3% vs. 33.9% 1 year OS liso-cel vs. SOC: 79.1% vs. 64.2%
PILOT (23)		#infused/#leukapheresed: 62/74	Leukapheresis to delivery: 24	80%; 54%	DOR: 12.1 PFS: 9 OS: NR	
TRANSCEND NHL 001 MCL cohort (24)		#infused/#leukapheresed: 88/104	Leukapheresis to delivery: 24.5	83%; 72%	DOR: 15.7 PFS: 15.3 OS: 18.2	
ZUMA-2 (25, 26)		#infused/#leukapheresed: 68/74	Leukapheresis to delivery: 16	93%; 67%	DOR: 28.2 PFS: 25.8 OS: 46.6	

CAR T cell, chimeric antigen receptor T cell; DLBCL, diffuse large B-cell lymphoma; MCL, mantle cell lymphoma; ORR, overall response rate; CR, complete response; SOC, standard of care; NR, not reached; HR, hazard ratio; CI, confidence interval; PFS, progression-free survival; OS, overall survival; DOR, duration of response.

Given the success of CAR T-cell therapy in the third-line setting, recent efforts have focused on CAR T-cell therapy earlier due to concerns about T-cell exhaustion with multiple lines of therapy and given the poor prognosis in primary refractory disease or early relapse. ZUMA-7 was a phase 3 trial that randomized patients with large B-cell lymphoma who had primary refractory disease or had relapsed within 12 months of first-line therapy 1:1 to receive (1) axi-cel or (2) standard-of-care (SOC) chemoimmunotherapy followed by high-dose chemotherapy and followed by autologous stem cell transplantation (ASCT) (16). Of the 359 patients who underwent randomization, 180 were randomized to axi-cel, with 170 patients actually receiving the infusion (94%). The baseline characteristics revealed a high-risk patient population as 74% had primary refractory disease and 17% were double-hit. Of the patients who underwent leukapheresis, the manufacturing success rate of axi-cel was 100%. The median time from leukapheresis to release of axi-cel to the investigator was 13 days. It is worth noting that only 36% of patients in the SOC arm went on to receive high-dose chemotherapy with ASCT, signifying that most patients continued to have a chemo-refractory disease. With a median follow-up of 24.9 months, the median event-free survival (EFS) was 8.3 months in the axi-cel arm vs. 2.0 months in the standard-of-care (SOC) arm. The 2-year EFS was 41% and 16% in the axi-cel and SOC arm, respectively. The ORR was 83% with 65% CR rate in the axi-cel arm compared with 50% ORR and 32% CR in the SOC arm. Given the clear improvement with CAR T-cell therapy in this high-risk patient population, axi-cel was approved in 2022 by the FDA for patients with large cell lymphoma that was primary refractory or relapsed within 1 year.

#### Tisagenlecleucel

Tisagenlecleucel (tisa-cel) is made by using a lentiviral vector and includes a CD8a transmembrane domain and a 4–1BB costimulatory domain. Tisa-cel was the second CAR T-cell product approved by the FDA for relapsed/refractory DLBCL, transformed follicular lymphoma, or high-grade B-cell lymphoma after failure of two lines of therapy following the results of the phase 2 JULIET trial (17). Of the 165 patients enrolled in the study, only 111 received infusion of tisa-cel (17). For 12 patients, tisa-cel was unable to be manufactured. The median time from enrollment to infusion was 54 days. Among the 93 patients with evaluable responses, ORR was 52%, with 40% achieving a CR. The 1-year PFS was 35%, with OS of 49% for patients who underwent infusion. Unlike the ZUMA-1 trial, the JULIET trial did allow patients to undergo bridging therapy which occurred in 92% of patients given the long period between leukapheresis and infusion of product. A longer follow-up study of JULIET showed a median OS of 11.1 months (18). However, median PFS and OS were not reached for those achieving CR at 3 and 6 months, also demonstrating the curative potential of tisa-cel.

Like axi-cel, there was interest in bringing tisa-cel to the second-line setting. The BELINDA trial was a phase 3 trial comparing tisa-cel *versus* SOC salvage chemo-immunotherapy followed by ASCT in patients with aggressive B-cell lymphoma that was refractory or relapsed within 12 months of first-line chemo-immunotherapy (19). Of the 322 patients who underwent leukapheresis and randomization, 162 were assigned to receive tisa-cel (83% received bridging therapy). The baseline characteristics revealed a high-risk patient population as two-thirds had a primary refractory disease. The manufacturing success rate of tisa-cel was 97% and was infused in 96% of patients assigned to the experimental arm. The median time from leukapheresis to tisa-cel infusion was 52 days (range of 31 to 135), which is significantly longer than axi-cel. Like the ZUMA-7 trial, a minority of patients (32%) in the SOC arm in the BELINDA trial underwent autologous stem cell transplant as most had a chemo-refractory disease. Interestingly, the response assessment was performed prior to infusion, and a progressive disease was noted to be higher in the patients randomized to receive tisa-cel compared with those assigned to the SOC (26% vs. 14%). The best ORR was 46% (28.4% CR) in the tisa-cel group and 42% (15% CR) in the SOC group at 12 weeks. EFS was not significantly different between the treatment arms, and the median EFS was 3 months in both groups. A major reason thought to contribute to the negative results is the long manufacturing time of tisa-cel, which translated to some patients not having adequate time to respond to tisa-cel at week 12 assessment. While the authors of the trial noted that some patients had a response at later time points in the absence of lymphoma-directed therapy—thus suggesting the efficacy of tisa-cel—unfortunately, failure to respond at week 12 was counted as a negative event per the trial’s definition of EFS. The long manufacturing also resulted in some patients becoming refractory to bridging therapy or worsening performance status by the time the product was delivered, which may have also contributed to worse outcomes in the tisa-cel arm. Additionally, after randomization, the tisa-cel arm had patients with a higher-risk disease as 26% had progressive disease pre-infusion compared to the 14% in the SOC arm, which may have also contributed to the negative results as some studies have noted that a higher disease burden was associated with a lower chance of long-term remissions with CAR T-cell therapy (27). To date, tisa-cel is only approved after two failed lines of therapy.

### Lisocabtagene maraleucel

Lisocabtagene maraleucel (liso-cel) is made by using a lentiviral vector and includes a CD28 transmembrane domain and a 4–1BB costimulatory domain. However, unlike axi-cel and tisa-cel, T cells are separated to CD4+ and CD8+ CAR T cells and infused to patients as a sequential infusion at equal target doses (28). Liso-cel was approved by the FDA following the results of the TRANSCEND NHL 001 study which evaluated the efficacy in patients with R/R DLBCL, high-grade B-cell lymphoma, transformed from indolent lymphoma, primary mediastinal B-cell lymphoma, and follicular lymphoma grade 3B following failure of two or more lines of treatment (20). Two-thirds of the study population were chemo-refractory. Of the 344 patients who underwent leukapheresis, only 294 received CAR T-cell product (of which 25 received a non-conforming product). In two patients, product was unable to be manufactured, and 33 patients died prior to receipt of CAR T-cell therapy, indicating the high-risk patient population. Bridging therapy (given to 59% of patients) was allowed. The median time from leukapheresis to availability for shipment was 24 days (range, 17–51), while the time to leukapheresis to infusion was 37 days (range, 27–224). Unlike the ZUMA-1 and JULIET trials, the patients with secondary CNS involvement were eligible (3% of patient population). ORR was 73%, and 53% achieved CR. The 1-year OS was 58% for the total population and not reached for those with CR. The efficacy of those who received a non-conforming product was similar to those who received liso-cel. Re-treatment with liso-cel occurred in 16 patients who relapsed after an initial response, but ORR was low at 19% and response to re-treatment was not durable. In the 2-year follow-up study, the median duration of response (DOR), PFS, and OS were 23.1, 6.8, and 27.3 months (21). However, the median OS of those who achieved a CR was 48.5 months, demonstrating the long-term remission of CAR T-cell therapy for large B-cell lymphoma.

Much like axi-cel and tisa-cel, liso-cel was also studied in the second-line setting for those patients with high-risk aggressive B-cell lymphoma with refractory disease. The TRANSFORM study was the liso-cel equivalent of the ZUMA-7 and BELINDA trials: a phase 3 study comparing liso-cel with SOC salvage chemoimmunotherapy followed by ASCT (22) in patients with large B-cell lymphoma with primary refractory disease or relapse within 1 year of first-line chemoimmunotherapy. This study also allowed crossover to receive liso-cel if patients in the SOC arm failed to achieve a response to salvage chemoimmunotherapy, had a progressive disease, or failed to achieve CR at 18 weeks post-randomization. A total of 184 patients were randomized (92 per arm), with nearly three quarters of patients having a refractory disease in each arm. All patients who were randomized underwent leukapheresis. Of the 92 patients who were in the liso-cel arm, 89 patients (97%) received liso-cel and one patient (1%) received a non-conforming product. There was manufacturing failure in one patient (1%). The median time from leukapheresis to product availability was 26 days (range, 19–84) and from leukapheresis to infusion was 36 days (range, 25–91). Bridging therapy was allowed and occurred in 63% of patients in the liso-cel group. Of the 92 patients in the SOC arm, only 46% achieved a response and received ASCT. A total of 50 of the 92 patients in the SOC were approved for crossover, 46 patients received liso-cel, and one received a non-conforming product. ORR was 86% (CR of 66%) in the liso-cel arm and 48% (CR of 39%) in the SOC arm. The median EFS was 10.1 months for liso-cel vs. 2.3 months for SOC with respective 12-month EFS of 44.5% and 23.7%. The 1-year PFS and OS was 52.3% and 79.1% for liso-cel and 33.9% and 64.2% for SOC, respectively. Given the efficacy of liso-cel over SOC, liso-cel is now approved by the FDA in the second-line setting for patients with primary refractory large B-cell lymphoma or relapse within 12 months of finishing frontline treatment.

Liso-cel is also approved for first relapses in patients with large B-cell lymphoma who are ineligible for ASCT due to age or other comorbidities based on the results of the phase 2 PILOT study (23). Of the 74 patients who underwent leukapheresis, 62 received CAR T cells (one of whom received a non-conforming product). Manufacturing success was 100%. The median time from leukapheresis to product release was 24 days, and the median time to infusion was 25.5 days. Bridging therapy was allowed and occurred in 52% of patients. Unlike the ZUMA-7, BELINDA, and TRANSFORM studies, the median age was much older at 74 years as the patients were transplant ineligible. About one-third of the patients were double or triple hit, and 54% were refractory to their last treatment. ORR was 80%, and 54% achieved CR. The median PFS was 9 months, and the median EFS was 7.2 months; the median OS was not reached. In those with CR, the median PFS was 22.6 months and the median OS was not reached. Given these efficacy results in a population who were not transplant eligible and thus without a curative option, the FDA approved liso-cel for transplant-ineligible patients with large B-cell lymphoma who failed in first-line therapy.

### Mantle cell lymphoma

#### Brexucabtagene autoleucel

Brexucabtagene autoleucel (brexu-cel, previously KTE-X19) is a CD-19-directed second-generation CAR T-cell therapy with the co-stimulatory domain CD28 but removes circulating CD19+ malignant B cells to reduce possible CAR T-cell activation and exhaustion (25). ZUMA-2 is a phase 2 trial which evaluated the efficacy of brexu-cel in patients with relapsed or refractory MCL who had received up to five previous therapies, including a monoclonal antibody, anthracycline- or bendamustine-based chemotherapy, and a BTK inhibitor. Bridging therapy with steroids or BTK inhibition was allowed and was received by 37% of patients. The primary end point was ORR. A total of 74 patients were enrolled. Brexu-cel was manufactured for 71 patients (96%) and administered to 68 patients. The median time from leukapheresis to product delivery was 16 days. In a pre-specified primary efficacy analysis of the first 60 treated patients who had at least 7 months of follow-up, 93% had an ORR as assessed by an independent radiologic review, with 67% having a complete response (CR). In the intention-to-treat analysis, 85% had an ORR; 59% had a CR. At a median follow-up of 12.3 months, 57% of the 60 patients in the primary efficacy analysis were in CR. At 12 months, the estimated progression-free survival and overall survival were 61% and 83%, respectively.

Importantly, these remarkable and durable remissions in ZUMA-2 were the same across all poor prognosis subgroups, including age >65, blastoid or pleomorphic variants, high Ki-67, TP53-mutated, and high MIPI score. These findings are salient for patients with TP53 mutations and blastoid or pleomorphic subtypes who traditionally have not had sustainable long-term therapeutic options (29, 30). At 3 years of follow-up of the ZUMA-2 study, the median duration of response was 28.2 months, with median PFS of 25.8 months and OS of 44.6 months (26). Brexu-cel was approved for the treatment of relapsed and refractory MCL following two lines of therapy by the FDA in July 2020 based on ZUMA-2. In retrospective studies looking at the real-world experience of brexu-cel in the standard-of-care practice in both the US and Europe, results and toxicities were similar to ZUMA-2 despite longer manufacturing times and a higher risk profile of patients who would not have been eligible for ZUMA-2 (31–33).

#### Lisocabtagene maraleucel

TRANSCEND NHL 001 was a seamless design study which evaluated the safety and efficacy of liso-cel in patients with relapsed or refractory large B-cell lymphomas and included a MCL cohort of patients after two prior lines of therapy including a BTK inhibitor, an alkylator, and an anti-CD20 monoclonal antibody (24). Bridging therapy was also allowed in this study. The primary endpoints were safety and ORR. Among the 104 patients with MCL who were leukapheresed, 88 patients received liso-cel, 83 patients were part of the efficacy analysis set, and 74 patients were part of the primary analysis set. A substantial number of these patients had high risk features, including 75% with a Ki67 greater than 30%, 23% with a TP53 mutation, 31% with blastoid morphology, and 8% with secondary CNS lymphoma at the time of infusion. The overall ORR was 86.5%, with 74.3% achieving a CR in the primary analysis set and was similar across all high-risk groups. The median duration of response (DOR) was 15.7 months, with a median PFS of 15.3 months and a median OS of 18.2 months at a median follow-up of 22.8–24 months (20). Based on these data, the FDA approval of liso-cel for MCL is expected in 2024.

### CNS involvement

CNS involvement represents a specific therapeutic challenge in the treatment of patients with relapsed and refractory aggressive B-cell lymphoma. The investigators were initially hesitant to include patients with CNS involvement in the landmark CAR T-cell therapy trials over concerns of a higher risk of neurological events. TRANSCEND NHL included a small number of large B-cell lymphoma patients with CNS involvement (20). More recently, several retrospective single-institution small case series of primary and secondary CNS DLBCL have shown safety and efficacy in the use of CAR T-cell therapy (34–38). A meta-analysis of 128 patients showed that those with primary CNS lymphoma had a CR of 56% and 37% remained in remission at 6 months (39). For those with secondary CNS lymphoma, CR was 47% and 37% were in remission at 6 months (39). CRS was 77% (13% grade 3 or higher) and 72% (11% grade 3 or higher) in primary CNS lymphoma and secondary CNS lymphoma, respectively. Immune-effector cell-associated neurotoxicity syndrome (ICANS) was experienced by 53% (18% grade 3 or higher) and 48% (26% grade 3 or higher), respectively. A second multicenter study of 61 patients with secondary CNS lymphoma who underwent CAR T-cell therapy had ORR of 68% and CR of 57% (40). The median PFS and OS were 3.3 and 7.6 months, respectively (40). CRS was 70% (16% grade 3 or higher), and ICANS was 57% (44% grade 3 or higher) (40). Recent case reports have specifically pointed to the safety of CAR T-therapy with brexu-cel in the treatment of MCL with CNS involvement, even in one patient whose primary presentation of CNS involvement was seizures and in another patient with blastoid MCL and neurolymphomatosis (41–43). Recently, a subgroup analysis of patients with secondary CNS lymphoma in TRANSCEND showed high response rates, with 86% of patients (6/7) achieving a CR (44). The ability of CAR T-cell therapy to be a potential therapeutic option for aggressive B-cell lymphoma patients with CNS involvement meets a clinical need which has, up until now, remained unmet.

## CAR T-cell toxicities

### Early toxicities

CAR T-cell therapies cause predictable toxicities following their administration. Two unique early toxicities are known as cytokine release syndrome (CRS) and immune-effector cell-associated neurotoxicity syndrome for which patients must be monitored within the first 30 days following receipt of therapy. CRS is the immune system’s response to the *in vivo* activation and expansion of the CAR T cells. CRS is the more common early toxicity and is graded on a scale of 1–4 per American Society for Transplantation and Cellular Therapy (ASTCT) (45). CRS manifests with fever, hypotension, and hypoxia. In its most severe forms, it requires intensive care monitoring and support due to end-organ damage. Ruling out infection in this immunocompromised population is also essential. Incidence and grading of CRS differ between CAR T-cell products. Axi-cel and brexu-cel have CD28 co-stimulation which results in rapid peak expansion of CAR T cells compared to those with 4–1BB co-stimulation (9). This often results in quicker onset and a higher incidence of CRS. High tumor burden is also associated with higher incidence and severity of CRS and neurotoxicity (46). Liso-cel and tisa-cel have 4–1BB co-stimulation, which results in more gradual expansion and longer persistence of T cells and have delayed CRS that are not as severe. Indeed this is what we see in clinical practice. In the ZUMA-1 trial (**Table 3**), CRS was observed in 93% of patients (13% were grade 3 or higher) at a median onset of 2 days with axi-cel (14). In the ZUMA-5 trial, CRS occurred in 91% (15% were grade 3 or higher) with a median onset of 2 days (25). In contrast, the incidence of CRS was 42% (2% grade 3 or higher) with a median onset of 5 days with liso-cel in the TRASCEND study (20). Similar incidences of CRS and its onset with liso-cel were reported in the TRANSFORM and PILOT studies (22, 23). In the JULIET study, CRS occurred in 58% of the patients (22% were grade 3 or higher) with a median onset of 3 days for tisa-cel (17). CRS is managed with supportive care such as anti-pyretics, fluids, and supplemental oxygen as well as early administration of steroids and tocilizumab, an IL-6 inhibitor. IL-6 is one of the many driving cytokines of this toxicity (47, 48). While close monitoring is required of patients experiencing CRS, it is reversible with early and appropriate treatment and supportive care and is experienced for a limited duration of time. In severe cases, vasopressors, mechanical ventilation, and high doses of steroids are used. Siltuximab, another IL-6 inhibitor that binds directly to IL-6 (unlike tocilizumab which binds to the IL-6 receptor) (49), is used off-label for tocilizumab-refractory CRS (50). Etanercept, infliximab, and anakinra have also been used off-label for tocilizumab-refractory CRS as tumor necrosis factor alpha (TNFα), and IL-1 also contributes to CRS (51–54).

**Table 3:** Toxicity of CAR T-cell therapy in aggressive B-cell lymphoma.

Trial	CRS (≥ grade 3)	ICANS (≥ grade 3)	Grade 5; other comments
ZUMA-1 (14, 15)	93% (13%) Median onset: 2 days	64% (28%) Median onset: 5 days	1% HLH/cardiac arrest
ZUMA-7 (16)	92% (6%) Median onset: 3 days	60% (21%) Median onset: 7 days	0%
JULIET (17, 18)	58% (22%) Median onset: 3 days	21% (12%) Median onset: 6 days	0%
BELINDA (19)	61% (5%) Median onset: 4 days	10% (2%) Median onset: 5 days	6%
TRANSCEND NHL 001 (20, 21)	42% (2%) Median onset: 5 days	30% (10%) Median onset: 9 days	6%
TRANSFORM (22)	49% (1%) Median onset: 5 days	12% (4%) Median onset: 11 days	14% (13) in liso-cel arm, 4 were from COVID 19, 7 due to disease progression
PILOT (23)	49% (2%)	31% (5%)	0%
TRANSCEND NHL 001 MCL cohort (24)	61% (1%) Median onset: 4 days	31% (9%) Median onset: 8 days	4% (infection, tumor lysis, unrelated cardiopulmonary arrest)
ZUMA-2 (25, 26)	91% (15%) Median onset: 2 days	63% (31%) Median onset: 7 days	3% (due to infections)

CAR T cell, chimeric antigen receptor T cell; DLBCL, diffuse large B-cell lymphoma; MCL, mantle cell lymphoma; CRS, cytokine release syndrome; ICANS, immune effector cell-associated neurotoxicity syndrome.

ICANS is the brain’s response to the exposure of cytokines from surrounding immune cells secondary to CAR T-cell activation and expansion. ICANS is generally less common than CRS and can manifests with a wide range of neurological symptoms, including tremor, headache, aphasia, inattention, confusion, somnolence, coma, and/or seizures in its most severe forms. It generally occurs after CRS symptoms. ICANS is graded on a scale of 1–4 using a standardized immune effector encephalopathy (ICE) scoring system which evaluates alterations in speech, orientation, handwriting, attention, and receptive aphasia and is traditionally effectively managed using steroids +/- levetiracetam prophylaxis (45). In the majority of cases, ICANS is reversible, though less severe symptoms can linger in approximately 10% of patients. Like CRS, the incidence and the severity of ICANS are higher and occur earlier with CAR T-cell products with C28 co-stimulation. ICANs occurred in 64% (28% were grade 3 or higher) of patients receiving axi-cel in ZUMA 1 trial (14) with a median onset of 5 days and with similar results in the ZUMA-7 study (16). In the ZUMA-5 study, the incidence of ICANS with brexu-cel was 64% (32% grade 3 or higher) with a median onset of 7 days (25). In contrast, liso-cel was associated with ICANs incidence of 30% (10% grade 3 or higher) with a median onset of 9 days in the TRANSCEND study (20) and with similar results in the MCL cohort (24) and in the PILOT study (23). The incidence of ICANS was far lower in the TRANSFORM study with liso-cel with incidence of 12% (2% grade 3 or higher) at a median onset of 11 days (22). ICANS occurred in 21% of patients (12% grade 3 or higher) with a median onset of 6 days in patients who received tisa-cel in the JULIET trial (17).

Optimizing prevention strategies for CRS and ICANS is an ongoing area of research. Recently, Park et al. published the interim results of their phase 2 study looking at the efficacy of prophylactic anakinra, a commercially available IL-1 receptor antagonist, in participants with LBCLs, including MCL receiving CD-19-directed CAR T-cell therapies (axi-cel, brexu-cel, or tisa-cel) (55). In this study, 74% of the participants experienced CRS, with 6.4% experiencing grade 3 or greater, and 19% of the participants experienced ICANS, with 9.7% experiencing grade 3 or greater. Of the participants receiving axi-cel and brexu-cel, ICANS occurred in 22% of the participants, with 11% experiencing grade 3 or greater compared to over 60% overall and 28%–31% greater than grade 3 reported in ZUMA-1 and ZUMA-2 trials (14, 25).

The rationale for the use of anakinra, a commercially available IL-1 inhibitor, is based on pre-clinical models in mice, trends observed in the CSF of patients experiencing ICANS, and the ability of IL-1 receptor inhibitors to cross the blood–brain barrier (56–60). In both pre-clinical murine models, the mice were treated with CAR T cells and clinically manifested CRS. Monocytes were the source of both IL-6 and IL-1 driving the CRS. While IL-6 blockade with tocilizumab was effective at controlling the manifestations of CRS, it was not protective of neurotoxicity and inflammation. IL-1 blockade, however, was effective at mitigating the manifestations of both CRS and ICANS (56). Similarly, the CSF of patients with acute lymphoblastic leukemia experiencing ICANS was high in specific cytokines, including both IL-6 and IL-1 (57). These early findings represent a potential option for effective ICANS prophylaxis, especially in high-risk patient groups such as high-risk MCL patients and those with bulky disease burden or CNS involvement.

### Late toxicities

The most common toxicities of CAR T-cell therapy are cytopenias. Indeed, at 1 month post-infusion, only 61%, 51%, and 33% of patients receiving CAR T-cell therapies were found to have recovered their hemoglobin, platelet, and neutrophil counts in an early retrospective study looking at hematological toxicity (61). Factors associated with a lower likelihood of hematopoietic recovery included baseline cytopenias, CAR construct, higher peak C-reactive protein and ferritin levels, and increasing-grade ICANS with a similar trend in CRS. Protracted cytopenias can cause significant co-morbidity to patients receiving CAR T-cell therapies.

The most morbid cytopenia is prolonged and severe neutropenia, which puts patients receiving CAR T-cell therapy at an increased risk for serious infection. Indeed advances in the management of both CRS and ICANS have led to fatal infections currently representing the most common cause of non-relapse mortality (NRM) in patients receiving this therapy (62, 63). All patients receive lymphodepleting chemotherapy prior to receipt of CAR T cells to provide an optimal environment for their expansion. This naturally leads to a transient period of cytopenia with expected recovery within 7–14 days post-chemotherapy. Protracted cytopenias, however, occur several weeks beyond this expected time frame and are felt to be due to immune dysregulation and inflammation occurring in the bone marrow following the administration of CAR T cells, though our understanding of this toxicity is evolving (63). Neutrophil recovery following infusion of CAR T-cells has been shown to exhibit quick, intermittent, or aplastic patterns (64, 65). The quick pattern shows sustained neutrophil recovery without any subsequent dips. The intermittent pattern shows neutrophil recovery followed by a second dip in neutrophil counts following day 21. Finally, the aplastic pattern shows continuous and severe neutropenia for greater than 14 days. Interestingly, an association between clinical outcomes and neutrophil recovery patterns has been found. The best clinical outcomes are associated to the intermittent neutrophil recovery pattern. The poorest clinical outcomes are associated to the aplastic neutrophil recovery pattern thought to be secondary to the presence of immune dysregulation which suppresses the expansion of CAR T cells (66).

In September 2023, the European Hematology Association/European Society for Blood and Marrow Transplantation (EBMT) released consensus grading and practice recommendations for immune effector cell-associated hematotoxicity (ICAHT) (67). ICAHT grading is based on the duration and severity of neutropenia. As part of the practice recommendations, the CAR-HEMATOTOX score is used to identify patients at a high risk of prolonged neutropenia and aplastic phenotype of neutrophil recovery (64). The score is calculated by looking at baseline bone marrow reserve (absolute neutrophil count, hemoglobin, and platelet count) and baseline inflammatory state (C-reactive protein and ferritin) prior to the receipt of lymphodepletion and places patients in either low risk or high risk categories. Based on this risk stratification, recommendations for anti-microbial prophylaxis, transfusion, and growth factor support have been suggested (68). The use of the CAR-HEMATOTOX score represents an important avenue to improve the supportive management of the infectious complications associated to CAR T-cell therapy. The association between clinical outcomes, baseline bone marrow reserve and inflammatory state, and hematological toxicity in patients receiving CAR T-cell therapy is an evolving area of research.

As more longitudinal experience is gained with CAR T-cell therapies, rare complications have emerged. While initially thought only to occur in conjunction with CRS, a life-threatening hemophagocytic lymphohistiocytosis (HLH)-like syndrome is increasingly being recognized post-CAR T-cell therapy. This entity often presenting as CRS is resolving or resolved and is believed to be associated to a protracted and exaggerated immune response which can cause end-organ damage. Current management strategies are derived from the expert opinion of those who have experiences this rare presentation and include the prompt initiation of anakinra and steroids with the addition of ruxolitinib or emapalumab if the case is progressively life-threatening (69). In addition, CAR T-cell therapies have recently been associated to a risk of secondary T-cell malignancies manifesting within 2 years of their receipt. Of the 22 cases known to the FDA as of December 2023, three had genetic sequencing performed, which detected the CAR transgene in the malignant clone, suggesting that the product was directly implicated in producing the cancer (70). Close monitoring of these rare but serious toxicities is warranted as well as the strategies to prevent them.

### Bridging therapy

The administration of CAR T-cell therapies poses unique challenges. CAR T-cell manufacturing, depending on the CAR T-cell product, can take several weeks to months to complete. Clinically, this means that there is a period of time where patients progressing on their last line of therapy must wait and remain stable until they can receive their CAR T cells. This period is supported by “bridging therapy” for disease control and can include steroids, chemotherapy, radiation, or targeted therapies. Given the aggressiveness of aggressive B-cell lymphoma and the limited therapeutic options, this poses a specific challenge to these patients. Manufacturing time and burden of disease at relapse are particularly salient to differences between the administration of cellular therapies in the clinical trial *versus* real-world setting (71). In the ZUMA-2 trial, bridging therapies were limited to steroids and BTK inhibitors (on patients already having progressed on BTK inhibition), and only 37% of patients required bridging, suggesting a population with less disease burden (25). In the benchmark retrospective studies looking at outcomes post-ibrutinib in the pre-CAR T cell era, 29.8–37.9% of patients progressing on BTK inhibitors never received subsequent therapies as they rapidly deteriorated and died (5, 6). Early signs of progression on BTK inhibition or suboptimal clinical response should prompt referral for CAR T-cell therapy in MCL. Even in the ZUMA-1, TRANSCEND NHL 001, and JULIET studies, a significant number of patients did not receive CAR T-cell therapy due to complications related to disease progression or death (14, 17, 20). While bridging therapy prior to CAR T cell varies, one retrospective review of 439 patients with 80 receiving bendamustine prior to leukapheresis was associated with lower ORR (53% vs. 72%) as well as shorter PFS (3.1 vs. 6.2 months) and OS (10.3 vs. 23.5 months) with CAR T-cell therapy (72). The authors of the study noted that bendamustine use within 9 months of leukapheresis was also associated with worse outcomes in terms of ORR, PFS, and OS with CAR T-cell therapy, suggesting that its use should be avoided in CAR T-cell eligible patients. Radiation has also been used as an effective bridging strategy in several retrospective studies (73, 74). Radiation is thought to work synergistically with CAR T-cell therapy by increasing the release of tumor-specific antigens, thus improving tumor recognition by immune cells as well as increasing the sensitivity of tumor to the cytotoxic effects by CAR T cells (75, 76).

Predictors of success and failure of CAR T-cell therapy can be patient, disease, or CAR T-cell product-related. Both patient fitness prior to therapy and the degree of tumor burden at cell infusion impact the efficacy of CAR T cells, making effective bridging and conditioning strategies a key factor in success treatment (77). In addition, the cellular starting material and T-cell fitness impact the cell manufacturing process and the efficacy of the product—for example, the presence of monocytes–reduces T-cell transduction and CAR T-cell expansion *in vitro (* 78).

### Consolidation with hematopoietic stem cell transplant

Currently, there is no data to support consolidation with hematopoietic stem cell transplant following CAR T-cell therapy. In the ZUMA 1 study, two patients who responded to axi-cel for DLBCL underwent allogeneic stem cell transplant (alloSCT) (14). In the long-term study, the median OS of those who achieved a CR was not reached (15), suggesting that axi-cel was potentially curative as majority of the patients did not receive consolidative transplant. AlloSCT has been used in those who had relapsed after CAR T-cell therapy. The American Society of Transplantation and Cellular Therapy (ASTCT) considers ASCT for consolidation for early-relapse DLBCL patients who achieve a PR or CR following salvage chemotherapy as a category B recommendation (79). They also consider CAR T-cell therapy as an acceptable alternative in the same patient population, also with a category B recommendation (79).

In one multi-center retrospective study, 88 patients underwent alloSCT following failure of CAR T-cell therapy (80) for DLBCL. The follow-up was short, with a median of 15 months, and the 1-year PFS and OS were 45% and 59%, respectively. The 1-year non-relapse mortality was high 22%, and the 1-year relapse/progression rate was 33%.

For MCL, only one patient who had a PR following brexu-cel underwent alloSCT (25); thus, the role of consolidative transplantation following CAR T-cell therapy is unknown. The ASTCT (American Society for Transplantation and Cellular Therapy), CIBMTR (Center for International Blood and Marrow Transplant Research), and EBMT (European Society for Blood and Marrow Transplantation) recommend alloSCT for MCL patients who relapse or progress following CAR T-cell therapy if they achieve CR or PR with subsequent lymphoma-directed therapies (81).

### Cost-effectiveness

While CAR T-cell therapies represent a paradigm shifting standard-of-care practice in the treatment of relapsed and refractory lymphomas with meaningful and prolonged remissions for patients, the resources required to manufacture these personalized products are significant, not to mention the burden on the patient. In one study of over 3,900 patients eligible for CAR T-cell therapy, over one-third traveled over an hour to the nearest academic center (82). Several cost-effectiveness analyses have been conducted to better understand the relationship between patient benefit and the economic impact of axi-cel, liso-cel, tisa-cel, and brexu-cel within North America and Europe for patients with R/R aggressive B-cell lymphoma (83–90).

The first study to look at the cost-effectiveness of CAR T-cell therapy used a decision analytic Markov model and assumed that, at 40% 5-year PFS, axi-cel increased the life expectancy by 8.2 years at $129,000/quality-adjusted life years (QALY) gained (87). However, at 30% 5-year PFS, axi-cel increased the life expectancy by 6.4 years at $159,000/QALY gained. The 5-year ZUMA 1 study showed a 5-year PFS of 31% (15). For tisa-cel, assuming 35% 5-year PFS, life expectancy would be increased by 4.6 years at $168,000/QALY gained, while the numbers were 3.4 years gained at $233,000/QALY gained assuming a 25% 5-year PFS. The authors determined that the prices of axi-cel and tisa-cel would need to be reduced to $250,000 and $200,000, respectively, or payment only for patients who achieve CR. However, at the time of analysis, fewer SOC options were available to R/R DLBCL patients. A later study did not find second-line CAR T-cell therapy to be cost-effective in DLBCL patients (88) at a willingness-to-pay threshold of $200,000/QALY. However, two other cost analyses did find CAR T-cell therapy to be cost-effective in the second-line setting (89, 90) at a willingness-to-pay threshold of $150,000 in both studies as these studies took into account less effective and newer but more expensive and indefinite salvage treatment options.

In contract, numerous studies have shown brexu-cel to be a cost-effective alternative to standard-of-care practice due to its benefit in health-related quality-of-life and incremental survival. There is no established standard-of-care therapy in the treatment of relapsed or refractory MCL following the use of a BTK inhibitor. Options include lenalidomide, bortezomib, venetoclax, other BTK inhibitors, and bendamustine-containing chemo-immunotherapy regimens. Accepted comparisons for survival in patients with relapsed or refractory MCL who progressed on BTK inhibition include the retrospective SCOLAR-2 study conducted in Europe and a large 2016 retrospective study by Martin et al. (5, 6).

In the cost-effectiveness analysis for brexu-cel in patients with relapsed/refractory MCL conducted in the United States, the population inputs and health state utilities were derived from the ZUMA-2 trial. The model assumed that patients whose disease had not progressed after 5 years experienced long-term remissions. In the analysis, the median survival was 9.71 years *versus* 2.13 years, estimated expected life years (LY) were 8.99 years vs. 4.47 years, and QALY were 7.39 years vs. 3.65 years for brexu-cel *versus* standard of care. The total cost for brexu-cel was $693,832 USD *versus* $574,263 USD for standard of care. The brexu-cel *versus* standard-of-care cost per QALY was $31 985 (83). The substantial LY and QALY benefit supports brexu-cel as a cost-effective therapy. The benefit was sustained in the cost-effectiveness analyses conducted in Canada, England, and Italy despite the total cost of brexu-cel and especially with the standard of care being significantly lower (84–86)—for example, in the cost-effectiveness analysis conducted in England, whose benchmark for standard of care was the SCHOLAR-2 study, the total cost of brexu-cel *versus* SOC was £385,765 *versus* £48,645. The brexu-cel versus SOC cost per QALY remained comparable with the findings in the US at £67,713 (85). These findings support the continued development of CAR T cell and other cellular therapies for patients with relapsed and refractory MCL.

### Comparison of CAR T-cell products

While there is only one CAR T-cell product for MCL currently, there are three for large B-cell lymphomas. The choice of product is chosen by the cellular therapy specialist and considers the impact of various factors such as manufacturing time, toxicities, and efficacy as well as patient-related factors such as co-morbidities, age, and tumor burden. While axi-cel is associated with a higher incidence and a higher grade of CRS and ICANS, the manufacturing time is significantly shorter and the manufacturing success rate is higher than that of liso-cel and tisa-cel (14, 17, 20). This may be a good option for the young, healthy patients with a high tumor burden and refractory disease where time is of essence with the caveat that toxicities may be high, whereas older, frailer patients with multiple co-morbidities with a lower tumor burden may benefit from liso-cel or tisa-cel due to their lower toxicity profile with the option of outpatient administration but at the cost of longer manufacturing time and increased chance of receiving a non-conforming product. While non-conforming products have been shown to have similar efficacy to lisa-cel in the TRANSCEND NHL study (20), patients often have to enroll in an expanded access protocol to receive their CAR T cells, thus further delaying the time between leukapheresis and infusion.

### Relapses after CAR T-cell therapy

Resistance to CAR T-cell therapies includes loss of CD19 antigen, new mutations or post-translational modifications in CD19, defective manufacturing of T cells, insufficient T cell expansion, changes to the cytokine milieu or functioning of CD4/CD8, upregulation of negative regulatory receptors, interaction between the tumor microenvironment on T-cell expansion, and impaired death receptor signaling (91, 92). Genomic profiling can uncover these mechanisms and develop strategies to mitigate them—for example, single-cell RNA sequencing and multiplex cytokine profiling on serial peripheral blood samples of patients treated with brexu-cel who eventually relapsed showed that the proportion of T cells, particularly cytotoxic T cells (CTLs), decreased. While TIGIT, LAG3, and CD96 were the most common checkpoint molecules expressed on exhausted CTLs and T cells, in general, only TIGIT significantly increased after relapse. CTLs expanded during remission and contracted at relapse with upregulated TIGIT expression. In addition, tumor cells acquired TIGIT expression (93). Co-targeting TIGIT during CAR T-cell therapy may serve as another avenue to prevent CAR T-cell relapse in MCL. In addition, the receptor tyrosine kinase-like orphan receptor 1 (ROR1) is expressed on MCL cells and has been shown to be particularly elevated in CAR T-cell relapsed MCL cells (94, 95). *In vitro*, an antibody–drug conjugate of ROR1 conjugated to monomethyl auristatin E, known as VLS-101, has induced tumor regression in MCL models of CAR T cell, ibrutinib, and venetoclax resistance (96). A phase 1 study of VLS-101 demonstrated safety and durable responses in patients with MCL, including those who have received prior BTK inhibitors and cellular therapies (97).

### Future directions

While the current CAR T-cell products have revolutionized the treatment of aggressive B-cell lymphomas, improvement of the current landscape is already occurring. A phase 2 trial of axi-cel in high-risk large B-cell lymphoma patients who failed to achieve a Deauville score of 3 or better after two cycles of frontline chemoimmunotherapy has shown remarkable results of 78% CR (ORR of 89%), with median EFS and PFS not reached (98). Third-generation CAR T-cell products have two co-stimulatory domains containing CD28 and 4–1BB, which have been shown to improve efficacy *in vitro* and in animal models *in vivo*, with human trials being underway (99). Additionally, bispecific CAR T cells (targeted against both CD19 and CD20) have also been made to counteract the loss of CD19 expression in some B-cell lymphoproliferative disorders (100). CRISPR/Cas9 technology is also being used to enhance the effectiveness of CAR T-cell therapy by modifying T cells to improve their persistence and efficacy by disrupting genes associated with T cell exhaustion (101). Finally, allogeneic CAR T-cell products from healthy donors offer the most excitement as these counteract the need for leukapheresis and long wait time for manufacturing and potential for re-treatment if necessary. The phase 1 study of anti-CD19 allogeneic CAR T-cell products of the ALLO-501 and ALLO-501A ALPHA studies administered in patients with large B-cell lymphoma with two failed lines of treatment demonstrated a promising ORR of 67% with CR of 58% (102).

## Conclusion

The success of CAR T-cell therapy in the treatment of patients with aggressive B-cell lymphomas is practice-changing and provides a needed, durable therapeutic option for many patients who historically would have had dismal outcomes. While work remains to be done to optimize the effectiveness and toxicity management of this novel therapeutic approach and better incorporate it into the most effective sequence of therapy, especially with the advent of bispecific antibodies with milder toxicity profiles, there is no doubt that cellular therapies have changed the paradigm with which aggressive B-cell lymphoma patients are treated.

## Author contributions

BH: Conceptualization, Supervision, Writing – original draft, Writing – review & editing. VK: Writing – original draft, Writing – review & editing. MP: Writing – original draft, Writing – review & editing.
